# Pitstop 2 Is a Potent Inhibitor of Clathrin-Independent Endocytosis

**DOI:** 10.1371/journal.pone.0045799

**Published:** 2012-09-21

**Authors:** Dipannita Dutta, Chad D. Williamson, Nelson B. Cole, Julie G. Donaldson

**Affiliations:** Laboratory of Cell Biology, National Heart, Lung and Blood Institute, National Institutes of Health, Bethesda, Maryland, United States of America; University of Birmingham, United Kingdom

## Abstract

Clathrin independent endocytosis (CIE) is a form of endocytosis present in all cells that mediates the entry of nutrients, macromolecules and membrane proteins into cells. When compared to clathrin-dependent endocytosis (CDE), however, much less is known about the machinery involved in forming CIE endosomes. One way to distinguish CIE from CDE has been to deplete cells of coat proteins involved in CDE such as clathrin or the dynamin GTPase, leading to a block of CDE but not CIE. A drawback of such genetic manipulations is that depletion of proteins important for mediating CDE over a period of days can have complex indirect effects on cellular function. The identification of chemical compounds that specifically and rapidly block CDE or CIE would facilitate the determination of whether a process involved CDE or CIE. To date, all of those compounds have targeted CDE. Dynasore and the dynoles specifically target and block dynamin activity thus inhibiting CDE but not most forms of CIE. Recently, a new compound called pitstop 2 was identified as an inhibitor of the interaction of amphiphysin with the amino terminal domain of clathrin, and shown to inhibit CDE in cells. Here we show that pitstop 2 is also a potent inhibitor of CIE. The effects of pitstop 2 are not restricted to inhibition of clathrin since knockdown of clathrin fails to rescue the inhibition of endocytosis of CIE proteins by the drug. Thus pitstop 2 has additional cellular targets besides the amino terminal domain of clathrin and thus cannot be used to distinguish CIE from CDE.

## Introduction

Cells use a variety of means to internalize extracellular material and plasma membrane (PM) by the general process of endocytosis. All cells use this process to deliver extracellular nutrients into the cell interior, recycle PM to other regions of the cell surface, and to degrade PM proteins and lipids. Clathrin-dependent endocytosis (CDE) is an efficient and selective process whereby PM proteins containing specific cytoplasmic sorting sequences are gathered by adaptor proteins into clathrin-coated pits, and then are severed from the PM with the assistance of the dynamin 2 GTPase. CDE is widely studied, whereas much less is known about clathrin-independent endocytosis (CIE) although there is evidence of CIE in many cell types and multiple pathways have been characterized [1], [2], [3]. CIE includes modes of internalization for glycolipid-binding toxins such as shiga and cholera toxin [4], for GPI-anchored proteins (CLIC/GEEC) [5], for the EGF receptor under certain conditions [6], and for a number of endogenous PM proteins involved in immune function, nutrient uptake, and cell-cell and cell-matrix interactions [7]. There is a growing list of membrane proteins entering mammalian cells by CIE and there is now good evidence that CIE exists in lower eukaryotes 8,9. The identification of selective inhibitors of CDE and CIE would greatly enhance the characterization of specific physiological functions of these endocytic processes.

Many approaches have been taken to inhibit CDE [10]. The expression of mutants of proteins involved in the clathrin machinery, such as Dynamin2-K44A [11], the carboxy terminus of AP180 [12], and clathrin hubs [13], has proven quite effective. More recently siRNA-mediated depletion of the clathrin heavy chain, subunits of the AP2 adaptor [14], and dynamin 2 [15] have abolished CDE in cells. The drawback of these genetic approaches is that they require days to take effect and may lead to many indirect effects or compensatory cellular responses that make interpretation of the findings sometimes difficult. Use of a number of acute cellular treatments including cytosol acidification and hypotonic treatment can be effective at blocking endocytosis of CDE cargo [10] but these treatments are non-specific and may also affect CIE.

**Figure 1:**
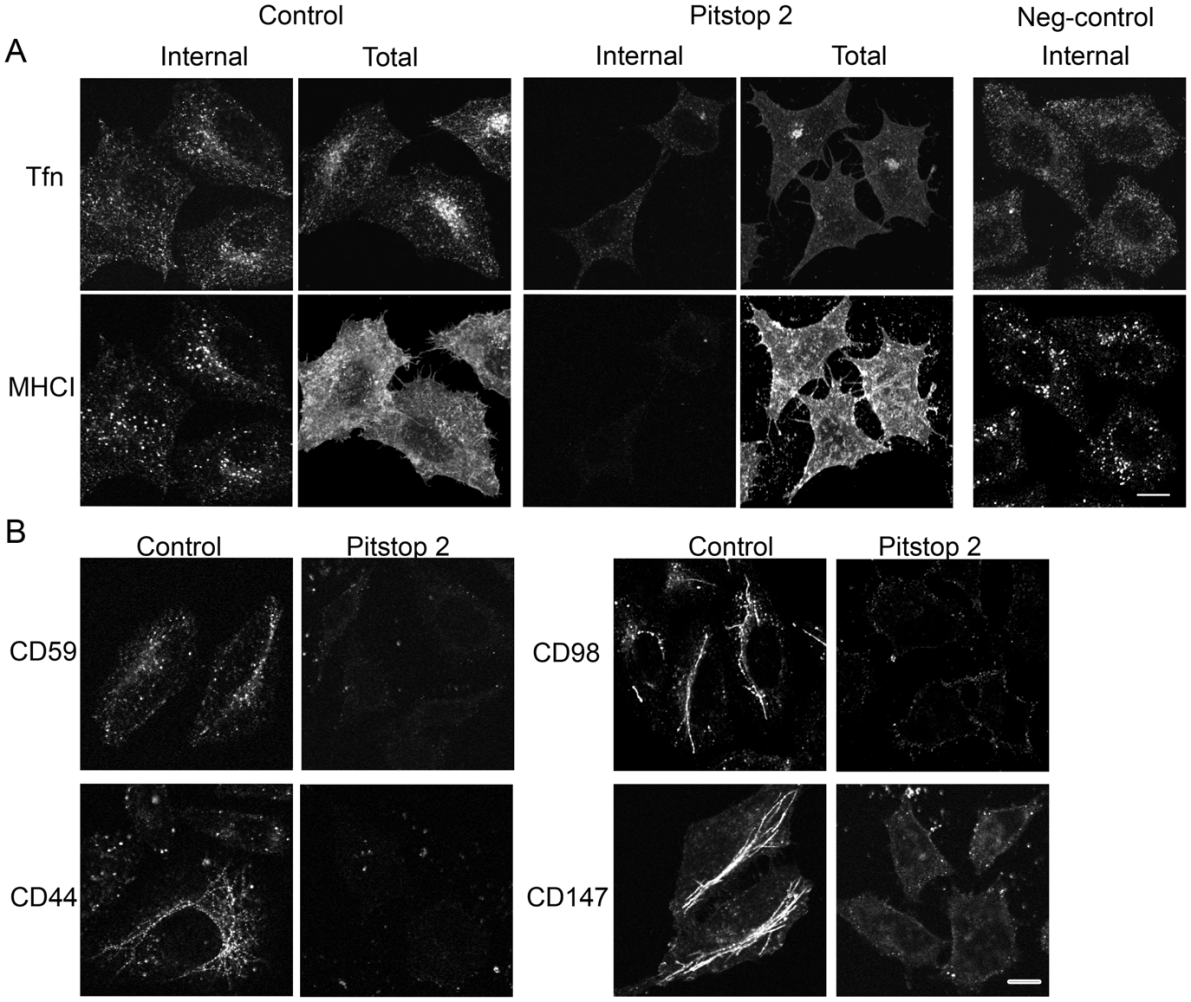
Pitstop 2 inhibits both clathrin-dependent and clathrin-independent endocytosis. (A) Hela cells were preincubated with DMSO (control) or 20 µM pitstop 2 or pitstop 2-negative control for 15 min. Cells were allowed to internalize Alexa594-Transferrin and antibodies to MHCI for 30 min at 37°C in the presence of DMSO or drugs. Surface bound antibodies were removed by low pH wash to visualize the internalized proteins (Internal) prior to fixation or cells were fixed immediately after the internalization to label for the internal and surface pools (Total) of the protein. Cells were then labeled with secondary antibodies to detect MHCI. (B) Cells preincubated with DMSO or 20 µM pitstop 2 were allowed to internalize antibodies directed towards CD59, CD44, CD98 and CD147 for 30 min and fixed. Surface bound antibodies were removed by acidic wash or blocked with unlabeled goat-anti-mouse IgG. Cells were then incubated with secondary antibodies to detect the internalized cargo proteins. The results shown are representative of three independent experiments. Bar, 10 µm.

Recently, new compounds that selectively target proteins involved in CDE have been identified with the promise that these could be used to acutely inhibit this process. These include compounds that specifically target dynamin such as dynasore [16] and the dynoles [17]. Since dynamin is required for all forms of CDE and is used in some forms of CIE [18], a compound that selectively targets clathrin was developed by Haucke and colleagues. This compound, named pitstop 2, was designed and shown to bind to and block interactions between the amino terminal domain of clathrin heavy chain and amphiphysin, one of many proteins shown to bind to this domain of clathrin [19]. In cells, pitstop 2 was shown to inhibit endocytosis of transferrin receptor, a CDE cargo protein, but not affect endocytosis of shiga toxin [19], which enters cells independently of clathrin [20].

We attempted to use pitstop to acutely block CDE in order to examine effects of blocking CDE on subsequent trafficking of endocytosed CIE cargo proteins. Surprisingly, we found that pitstop 2 potently blocks endocytosis of endogenous proteins normally entering cells by CIE.

**Figure 2:**
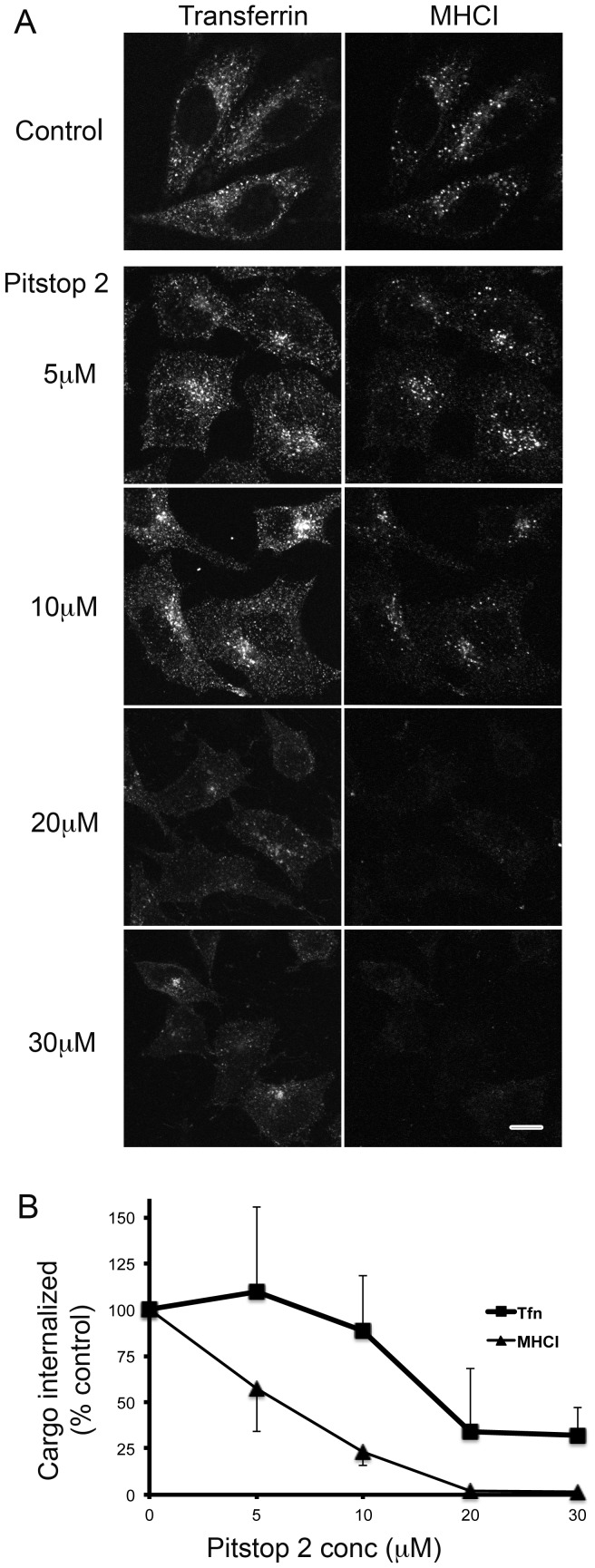
Pitstop 2 inhibits clathrin-independent endocytosis in a dose-dependent manner. (A) Hela cells were preincubated with DMSO (0.1%) or different doses of pitstop 2 ranging from 5 µM to 30 µM. Cells were then allowed to internalize Alexa594-Transferrin and antibodies to MHCI in the presence or absence of the drug for 30 min and then processed as described in Figure 1. Bars, 10 µm. (B) Total integrated fluorescence intensity of internalized Tfn and MHCI was quantified using Metamorph Software as described in Materials and Methods. The values of the different doses of pitstop 2 were then normalized to DMSO controls (set to 100%). Quantification was done for 60 cells at each dose and the error bars represent the standard deviation from the mean. P-values were calculated from the raw data and compared between DMSO and different doses of pitstop 2. Inhibition of MHCI uptake with pitstop was statistically significant for all doses with p-value <0.001 except for 5 µM pitstop 2 with p-value <0.02. Tfn uptake was also inhibited at 20 and 30 µM with p<0.001. The images and quantification shown are from one experiment; similar results were obtained in two additional experiments.

## Materials and Methods

### Cells, Reagents and Antibodies

Hela and COS-7 cells were cultured in Dulbecco’s modified Eagle’s medium (DMEM) containing 10% fetal bovine serum (FBS) at 37°C with 5% CO_2_. BEAS-2B cells were grown in low glucose DMEM containing 10% fetal bovine serum. Pitstop 2 and pitstop 2-negative control were purchased from Abcam. Monoclonal antibodies directed towards MHCI (clone w6/32), CD59 (clone p282/H19), CD44 (clone BJ18), CD98 (clone MEM-108) and CD147 (clone HIM6) were from Biolegend. Alexa 594-conjugated Transferrin and Alexa 488-conjugated Transferrin were purchased from Invitrogen. BG-Alexa 488 is from New England Biolabs. Alexa 568-conjugated Shiga toxin was a generous gift from Dr. Olga Kovbasnjuk (Johns Hopkins Medical School).

**Figure 3:**
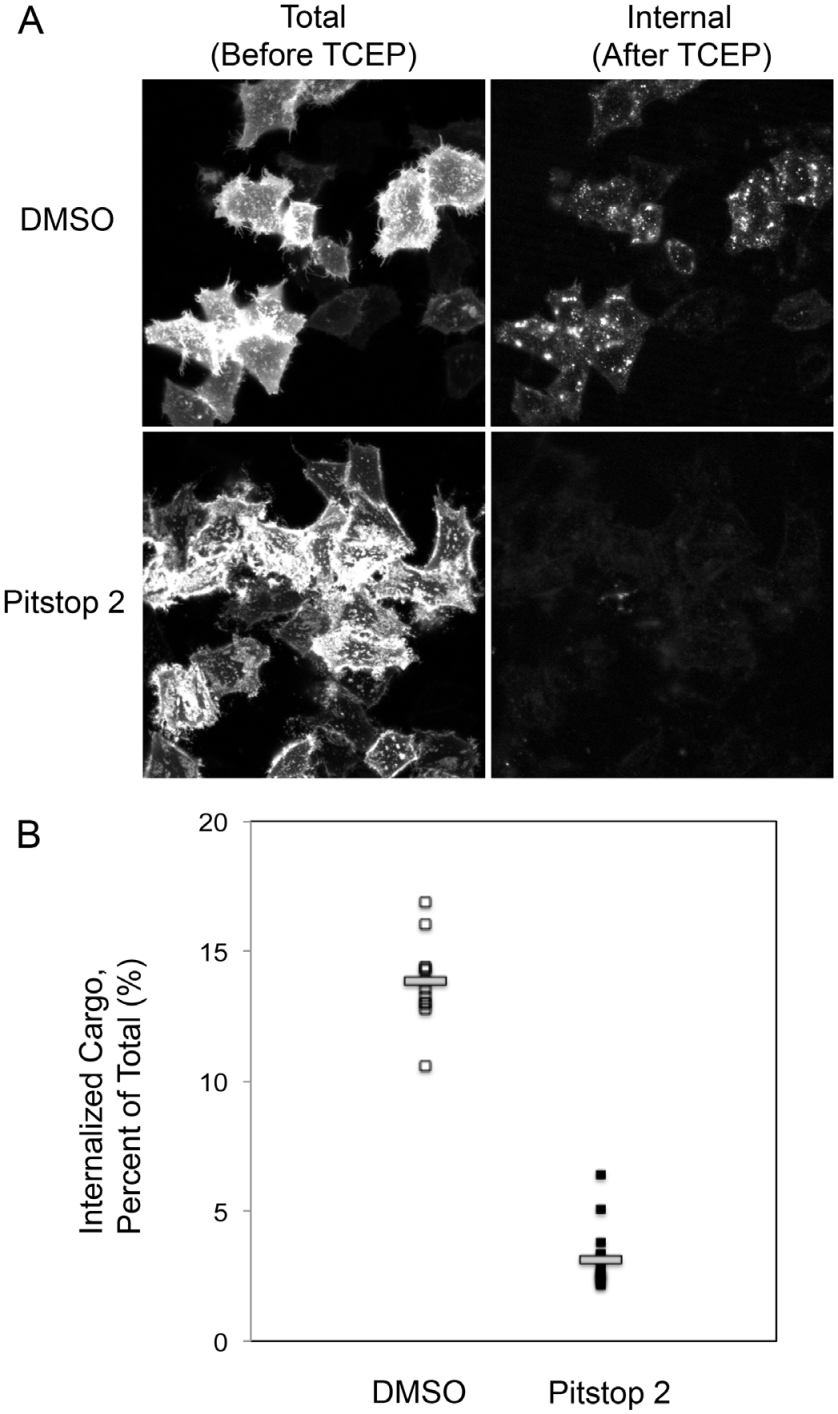
Pitstop 2 inhibits SNAP-Tac internalization in transfected HeLa cells. (A) HeLa cells transiently transfected with SNAP-Tac were preincubated for 10 min with either DMSO (control, top panels) or 20 µM pitstop 2 (bottom panels). Cells were then exposed to BG-S-S-594 and allowed to internalize for 30 minutes at 37**°**C in the presence or absence of pitstop 2. Cells were then washed with PBS, overlaid with Phenol Red-free DMEM containing 25 mM HEPES, and imaged live. Images in the left column were taken before the addition of TCEP, and represent total labeled SNAP-Tac protein (surface + internalized protein). Images of the same cells taken one min after TCEP treatment are shown in the right column, and represent the internalized pool of SNAP-Tac whose BG-S-S-594 label is protected from TCEP cleavage. (B) Percent internalized SNAP-Tac was calculated for individual cells displayed in (A) under either DMSO treatment or pitstop 2 treatment conditions. Small squares represent calculations of individual cells (12 cells measured for DMSO, 16 cells measured for pitstop 2 treatment). Horizontal bars represent the mean percent internalization for each group of cells (13.8% +/−1.6% for DMSO, 3.1% +/−1.1% for pitstop 2). This graph shows the quantification of a single endocytosis experiment. Data pooled from at least five matched sets (DMSO and pitstop 2) of comparable experiments and using two different pitstop 2 stocks demonstrated a similar inhibition of SNAP-Tac internalization in the presence of pitstop 2.

### RNA Interference and Constructs

Oligonucleotides (siRNA) directed towards the μ2 subunit of AP2 (5′-GUGGAUGCCUUUCGGGUCAuu-3′) and towards clathrin heavy chain (CHC) (5′-UAAUCCAAUUCGAAGACCAAUuu-3′) [14] were purchased from Dharmacon Thermo Scientific. Hela cells were plated in 35 mm dishes and transfected with each siRNA (200 pmol) using Lipofectamine 2000 (Invitrogen) using the manufacturer’s instructions. After 48 h, cells withμ2 siRNA were transfected again with siRNA and used in the experiments 48 h after the second transfection. For CHC siRNA, cells were transfected again 72 hours after first transfection and then used in the experiments 72 hours after the second transfection.

**Figure 4:**
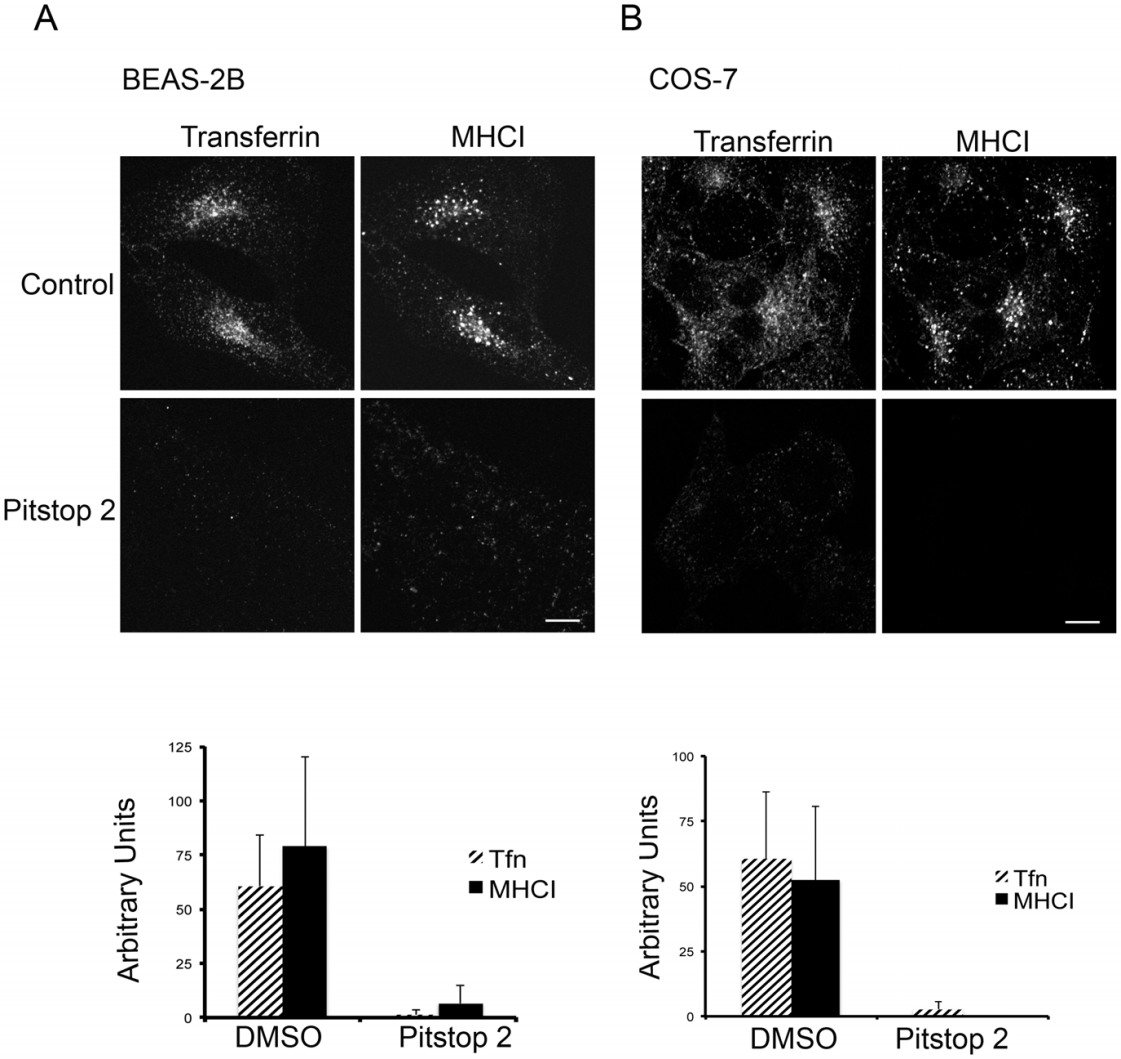
Pitstop 2 inhibits MHCI uptake in other cells lines. (A) BEAS-2B cells were preincubated with DMSO or 20 µM pitstop 2. Cells were then incubated with Alexa-594 Tfn and antibodies to MHCI in the presence of DMSO or the drugs for 30 min and then processed as described in Figure 2. Lower panel represents the quantification of the integrated intensity of internalized proteins (Tfn and MHCI). The total integrated intensity of each cell was quantified as described in Materials and Methods. The mean fluorescence intensity, as arbitrary units, is plotted with standard deviation for 50 cells in both control and pitstop-treated cells. Both Tfn and MHCI internalization were inhibited at 20 µM pitstop 2 (p<0.001). (B) COS-7 cells were allowed to internalize Alexa-488 Tfn and antibodies to MHCI in the absence or presence of pitstop 2, and quanitified as described in (A). Statistical analysis done for 50 cells with p<0.01 for Tfn inhibition and p<0.001 for MHCI inhibition. The images and quantification shown are from one experiment and similar to that observed in 2 additional experiments. Bars, 10 µm.

### Immunofluorescence and Antibody Internalization

Cells were seeded on cover slips overnight and then placed in serum-free media for 1 h prior to the experiment. Cells were treated with 20 µM pitstop 2 dissolved in serum-free media containing 10 mM Hepes for 15 min at 37°C prior to internalization assays. Control cells were treated with DMSO (0.1%) dissolved in DMEM. For cargo internalization assays, cells were incubated in media with or without the drug in the presence of 5–10 µg/ml antibodies directed toward the cargo protein at 37°C for 30 min to allow endocytosis of the antibody. Transferrin was allowed to internalize for 10 min in the presence or absence of the drug. After internalization, surface antibody was removed by low pH acid wash (0.5% acetic acid in 0.5 M NaCl for 30 s) before fixation or cells were incubated with unlabeled goat-anti mouse IgG in the absence of saponin. Internalized antibody was monitored by incubation with secondary antibodies in the presence of saponin. Images were obtained using a Zeiss 510 LSM confocal microscope. For Shiga toxin internalization, cells pretreated with drug or DMSO were incubated with 140 ng/ml Alexa568 labeled Shiga toxin at 37°C for 30 min prior to fixation. Quantification of images was done using Metamorph ×32 software. Fluorescence of individual cells was quantified separately, using inclusive threshold positioned at the right base of the histogram bar of the control cells. The same threshold values were used for the different doses of pitstop 2 for each experiment and total integrated fluorescence intensity was calculated for each individual cell.

**Figure 5:**
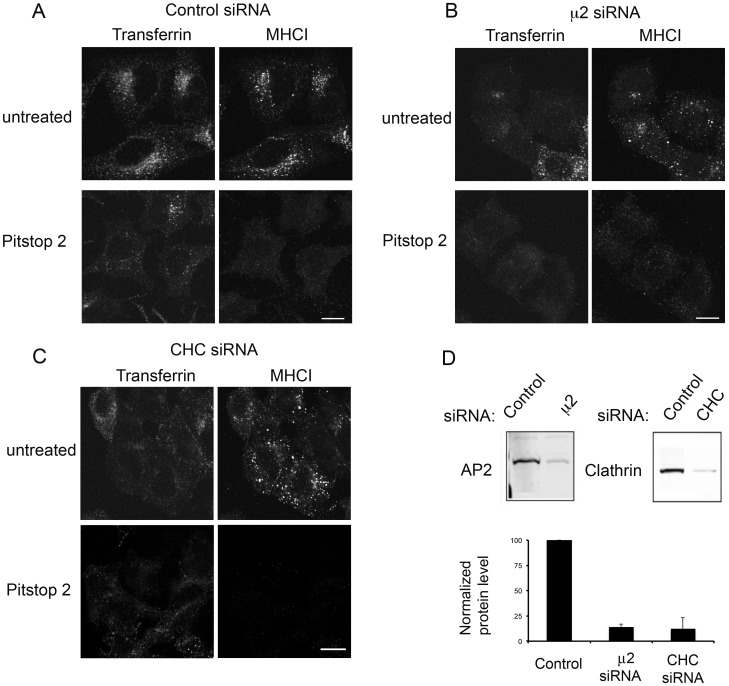
Pitstop 2 inhibition is not dependent upon AP2 or clathrin. Hela cells were treated with control siRNA (A) or siRNA to deplete cells of μ2 subunit of AP2 (B) or clathrin heavy chains (C) as described in Materials and Methods. (A–C) Cells were preincubated with DMSO or 20 µM pitstop 2 for 15 min. Alexa488-Tfn and antibodies to MHCI were allowed to internalize for 30 min and processed as in Figure 1. Bars, 10 µm. (D) Detergent lysates of Hela cells treated with control siRNA, μ2 siRNA or CHC siRNA were separated by SDS-PAGE and immunoblotted with antibodies against proteins indicated at the left. The gel shows the level of knock-down from one representative experiment. The means and standard deviation of three separate experiments are plotted in the lower panel showing levels to be 14% and 12% of control levels for μ2 and clathrin heavy chain, respectively.

### Construction of SNAP-Tac and Quantification of Internalization of BG-labeled SNAP-Tac in Live Cells

The signal peptide for hen egg lysozyme (MRSLLILVLCFLPLAALG) was introduced just prior to the second amino acid of the SNAP tag (DKDCEMKR…). Following the SNAP-tag [21], a (GGGGS)_2_ linker was introduced, followed by the extracellular, transmembrane and cytoplasmic tail domains of the alpha chain of the human IL2 receptor (Tac) (ELCDDD…KSRRTI stop). This construct was in the mammalian expression vector pcDNA3.1 and generated by standard PCR and cloning techniques.

**Figure 6:**
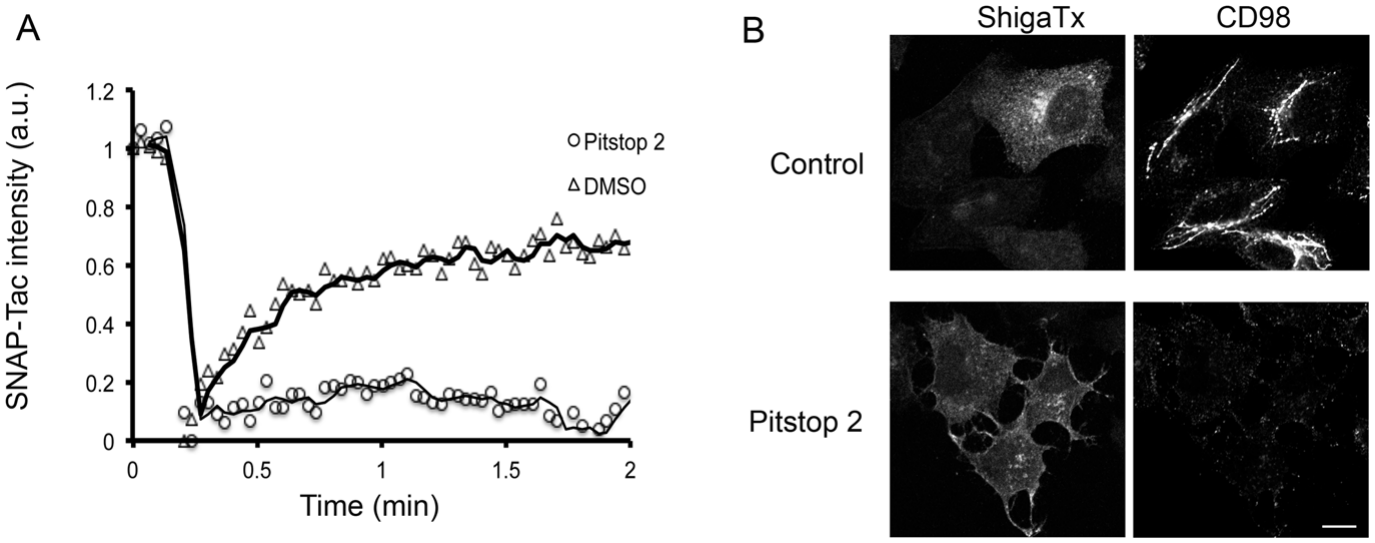
Pitstop 2 affects PM surface dynamics but shiga toxin entry is not blocked. (A) Hela cells expressing SNAP-Tac were preincubated with DMSO or 20 µM pitstop 2 for 15 min. Cells were labeled with BG-488 for 30 min at 37°C in the presence or absence of the drug and then imaged in the confocal microscope. Fluorescence recovery after photobleaching (FRAP) of surface SNAP-Tac was performed. Images were taken at 2 sec intervals, starting 10 sec before photobleaching, followed by imaging for a total time of 2 min after photobleaching. Corrected relative fluorescence intensities of surface SNAP-Tac in the FRAP region is represented in the graph. The graph was representative of 15 individual FRAP analyses from 2 independent experiments. (B) Hela cells pretreated with DMSO or pitstop 2 were allowed to internalize 568-labeled shiga toxin (140 ng/ml) and antibodies to CD98 for 30 min. Cells were fixed and labeled with 488-labeled secondary antibodies to detect internalized CD98. This experiment was repeated 2 additional times. Bars, 10 µm.

HeLa cells were seeded on to 8-well Lab-Tek chambered coverglass (ThermoScientific), and transiently transfected with SNAP-Tac plasmid using Fugene 9.0 reagent (Roche) following the manufacturer’s instructions. Twenty-four hours later, transfected cells were placed in serum-free media for 30 min, then treated with 20 µM pitstop 2 dissolved in serum-free media containing 0.1% DMSO for 10 min at 37**°**C. Control cells were treated with serum-free media containing 0.1% DMSO vector alone, also for 10 min at 37**°**C. Cell surface SNAP-Tac was labeled with an O^6^-benzylguanine probe fused via a cleavable disulfide linker to Alexa Fluor 594 (BG-S-S-594) [22], and incubated for 30 min at 37**°**C to allow endocytosis. BG-S-S-594 (from a 5 mM stock) was diluted 1∶2,500 in either pitstop 2 containing media, or control (DMSO) media, for SNAP-Tac cargo labeling. After 30 min of uptake, cells were quickly rinsed 4 times with 1X PBS, overlaid with Phenol Red-free DMEM (GIBCO) containing 25 mM HEPES, and immediately imaged. Image acquisition was as previously described [22]. Briefly, images were collected on a Zeiss 510 LSM confocal microscope with stage heated to 37**°**C by a Zeiss XL-3 environmental chamber, using a 40x Plan-Neofluor 1.3 (NA = 1.3) objective with the pinhole wide open. After taking an initial image, the cell impermeable reducing agent, tris (2-carboxyethyl)phosphine (TCEP) was directly added to cells, to a final concentration of 10 mM, to release the fluorophore from the BG-labeled SNAP-Tac on the plasma membrane. Another image was then taken 1 min after TCEP addition, revealing the internalized pool of SNAP-Tac cargo.

Metamorph ×32 software was utilized to calculate total fluorescent intensity measurements from each image. Individual cells were quantified separately, both before and 1 min after TCEP [22]. Percent internalization for each individual cell was then calculated by dividing the total fluorescent intensity measured at one minute after TCEP treatment by the total fluorescent intensity of the same cell measured just prior to TCEP treatment.

### FRAP Analysis

For live cell microscopy, Hela cells expressing SNAP-Tac were used. Transiently transfected cells were placed in serum-free media for 1 h. Cells were treated with 20 µM pitstop 2 for 15 min at 37°C. Surface SNAP-Tac was then labeled with BG-Alexa 488 for 15 min at 37°C in the presence or absence of the drug. The probe was washed with PBS and cells were imaged in DMEM without phenol red. Fluorescence recovery after photobleaching (FRAP) experiments were performed immediately. Images were taken at 2 sec intervals, starting 10 sec before photobleaching, followed by imaging for a total time of 2 min after photobleaching. Fluorescence of 488-labeled SNAP-Tac was analyzed in the photobleached region of the plasma membrane with time. Intensity values were corrected by analyzing the fluorescence intensity in a non-bleached control area.

## Results and Discussion

CDE and CIE can be observed in HeLa cells by monitoring endocytosis of labeled transferrin and an antibody to the Major Histocompatibility Complex Class I protein (MHCI), respectively. After 30 min of endocytosis, internalized transferrin and MHCI partially colocalized in the juxtanuclear regions (Fig. 1A, Control, Internal) and MHCI was also observed in some recycling tubules as described reported [23], 24. Similar to what has previously been reported [19], treatment of cells with 20 µM pitstop 2 led to a block in internalization of transferrin receptor compared to untreated cells (control) or cells treated with the negative control of pitstop 2 that was provided by the manufacturer (Fig. 1A). However, internalization of MHCI was also inhibited (Fig. 1A, Pitstop 2, Internal). Although endocytosis of MHCI was inhibited by Pitstop 2, the antibody was still capable of binding to the surface of cells as shown by imaging the total cell-associated fluorescence (Total) in control and Pitstop 2 treated cells (Fig. 1A). Endocytosis of other CIE cargo proteins was examined in the presence of pitstop 2. Internalization of CD59, a GPI-anchored protein with a trafficking itinerary similar to MHCI [7], [25], was also blocked by pitstop 2 (Fig. 1B). Three additional cargo proteins (CD44, CD98 and CD147), which enter cells by CIE but take an alternative itinerary from that of MHCI and CD59 once inside the cell [7], were also examined. Treatment with pitstop 2 blocked endocytosis of these proteins, while in untreated cells, endocytosed CD44, CD98 and CD147 were observed in tubular recycling endosomes (Fig. 1B), as previously observed [7]. The block in endocytosis induced by pitstop 2 was observed at shorter times (10 min) of internalization and could be reversed after 1 h of drug removal (data not shown).

The potent activity of pitstop 2 in blocking CIE was unexpected so we monitored its activity towards inhibiting transferrin and MHCI internalization with increasing doses of the compound (from 5–30 µM). In HeLa cells we found that endocytosis of MHCI appeared to be somewhat more sensitive to the action of pitstop 2 than that of transferrin (Fig. 2A). We also noticed that even at high doses of pitstop, some transferrin still enters cells. Quantification of internalization of transferrin and MHCI revealed a shift in the dose-response curve with half-maximal inhibition for MHCI at around 6 µM and for transferrin around 18 µM (Fig. 2B).

To further demonstrate that pitstop 2 blocks endocytosis of CIE cargo proteins, we turned to using a SNAP-tagged protein to quantify internalization in living cells. We recently developed a modification of labeling SNAP-tagged cell surface proteins using a releasable fluorescent tag on the benzylguanine (BG) ligand [22]. We transfected HeLa cells with a chimeric cargo protein consisting of the SNAP protein [21] attached to the extracellular portion of Tac, the IL2 receptor α-subunit. Tac enters cells by CIE and follows an intracellular itinerary similar to that of MHCI [23]. Cells expressing SNAP-Tac were labeled with BG-S-S-594 and allowed to internalize for 30 min in the absence and presence of pitstop 2. Cells were then imaged live and fluorescence quantified prior to (Total) and then 1 min after (Internal) addition of a cell-impermeable reducing agent (TCEP) that cleaves the 594 label from the surface [22]. This method allows for cell-by-cell quanitification of endocytosis. Pitstop 2 treatment reduced internalization of SNAP-Tac as compared to DMSO controls (Fig. 3A). The individual amounts internalized for each cell measured are plotted in Fig. 3B and clearly show a block in endocytosis in pitstop-treated cells. Furthermore, a similar amount of surface labeling with BG-S-S-594 was observed in control and pitstop-treated cells (Fig. 3A Total), indicating that pitstop did not interfere with BG binding to SNAP-Tac.

Next, we examined the effect of pitstop 2 on internalization of transferrin and MHCI in two other human cell lines. In both BEAS-2B, a lung epithelial cell line (Fig. 4A), and in COS-7 cells (Fig. 4B) inhibition by pitstop of transferrin and MHCI internalization was also observed. We did note, however, that in these cell lines, endocytosis of both transferrin and MHCI appeared to be blocked by pitstop 2 with similar potencies (not shown).

The shift in the dose-response curve observed in HeLa cells suggests that CIE may be more sensitive to the drug than CDE, raising the possibility that pitstop 2 has additional cellular targets besides the clathrin N-terminal domain. Alternatively, the equal sensitivity of CIE and CDE to pitstop 2 inhibition in COS-7 and BEAS-2B cells might suggest that pitstop 2 is acting in all cases through its effect in blocking the clathrin N-terminal domain. To examine whether pitstop 2 is inhibiting CIE through its effects on the clathrin N-terminal domain, we looked at transferrin and MHCI endocytosis in cells depleted of clathrin heavy chain or the μ2 subunit of the adaptor protein complex AP2 (Fig. 5D), both of which were depleted to approximately 12 and 14% of control levels, respectively. Depletion of the μ2 subunit of AP2 (Fig. 5B) or of clathrin heavy chain (Fig. 5C) by siRNA results in a block in transferrin endocytosis in most cells while endocytosis of MHCI by CIE is not affected (Fig. 5B and C, top rows). The addition of pitstop 2 to the μ2 and clathrin heavy chain depleted cells still led to a block in endocytosis of MHCI (Fig. 5B and C bottom row), suggesting that pitstop is blocking CIE through a site independent of clathrin.

To gain further insight into how this compound might be blocking CIE, a process that occurs independently of clathrin and dynamin but is sensitive to PM cholesterol levels [25], we asked whether mobility of cargo proteins entering cells by CIE might be affected by pitstop 2. To do this, we labeled cells expressing SNAP-Tac with the non-releasable probe, Alexa 488-conjugated BG ligand, and then imaged the cells live before and after photobleaching. In control cells treated with DMSO, surface fluorescence recovered with a t1/2 of approximately 30 sec (Fig. 6A). In contrast, there was no recovery of fluorescence for the duration of the experiment in cells treated with pitstop 2, suggesting that most of the PM SNAP-Tac was immobile (Fig. 6A). This dramatic change in surface mobility was also observed for GFP-labeled H-Ras (data not shown), a marker for the CIE endosomal membrane system [26]. A similar “freezing” of the clathrin and AP2 coat complexes with pitstop 2 was also observed in the original characterization of the compound [19], suggesting a striking target at the PM that may cause an inhibitory effect for most endocytic events or a general global alteration of PM structure. On the other hand, we did observe that endocytosis of shiga toxin still occurred in cells treated with pitstop 2 (Fig. 6B) as was previously reported [19], although the amount of shiga toxin internalized was less than in controls. Shiga toxin may be more resistant to pitstop as compared to other endogenous CIE cargo proteins due to its ability to bind to and cluster Gb3 glycolipid, forming a tubular invaginated entry structure into cells [20].

Taken together, our findings demonstrate that pitstop 2 cannot be used to determine that a protein enters cells by CDE since it blocks CIE as effectively as CDE. This effect, observed for many endogenous cargo proteins and in all human cell lines examined, is due to a second site of action for the compound since it still inhibits CIE in cells where clathrin has been depleted. This second site of action may explain some of the unusual behavior of cells treated with pitstop as pointed out by Lemmon and Traub [27]. It provides a cautionary tale for the in vivo application of “specific” small molecule inhibitors developed through chemical design as this approach cannot exclude second sites of action in living cells.
